# Incidence of extubation failure and its predictors among adult patients in intensive care unit of low-resource setting: A prospective observational study

**DOI:** 10.1371/journal.pone.0277915

**Published:** 2022-11-17

**Authors:** Natnael Kifle, Dereje Zewdu, Bisrat Abebe, Temesgen Tantu, Mekete Wondwosen, Yirgalem Hailu, Girma Bekele, Meron Woldetensay

**Affiliations:** 1 Department of Anesthesiology and Critical Care, Addis Ababa University, Addis Ababa, Ethiopia; 2 Department of Anesthesia, College of Medicine and Health Science, Wolkite University, Wolkite, Ethiopia; 3 Department of Anesthesiology and Critical Care, College of Medicine and Health Science, Wolaita Sodo University, Wolaita Sodo, Ethiopia; 4 Department of Obstetrics and Gynecology, College of Medicine and Health Science, Wolkite University, Wolkite, Ethiopia; 5 Department of Surgery, College of Medicine and Health Science, Wolkite University, Wolkite, Ethiopia; 6 Department of Internal Medicine, College of Medicine and Health Science, Wolkite University, Wolkite, Ethiopia; Shiraz University of Medical Sciences, ISLAMIC REPUBLIC OF IRAN

## Abstract

**Background:**

Previous studies have found an association between various predictors and extubation failure (EF) in intensive care units (ICUs). However, this problem remains unexplored in low-resource settings, where predicting the extubation outcomes are more challenging. This study investigates the incidence of EF and its predictors among patients who received mechanical ventilation (MV).

**Methods:**

This is a prospective observational study of 123 patients’ ≥ 18 years of age receiving MV for ≥ 48 hours and tolerated spontaneous breathing trials (SBTs) in the ICU of a low-resource setting. We collected data on the baseline characteristics and clinical profiles before and after SBTs. Patients were categorized into extubation failure (EF) and extubation success (ES) groups. Multivariate logistic regression analyses were performed to identify independent predictors for EF. A p-value < 0.05 is considered statistically significant.

**Results:**

We included 123 patients, and 42 (34.15%) had developed EF. The identified predictors for EF: Moderate to copious secretions (adjusted odds ratio [AOR]: 3.483 [95% confidence interval [CI] 1.10–11.4]), age > 60 years of age ([AOR]: 4.157 [95% CI 1.38–12.48]), and prolonged duration of MV ≥ 10 days ([AOR]: 4.77 [95% CI 1.55–14.66]).

**Conclusion:**

Moderate to copious secretions, patients > 60 years of age, and prolonged duration of MV ≥ 10 days were the best predictors of EF. Based on our findings, we recommend that the identified predictors could help in the decision-making process of extubation from MV.

## Introduction

Invasive mechanical ventilation (IMV) is a life support intervention among critically ill patients in intensive care units (ICUs). The decision of extubation is considered as soon as a patient meets weaning criteria and successfully passes a spontaneous breathing trial (SBT). However, extubation of ventilated patients is not a risk-free procedure. About 5–20% of planned extubations ultimately fail in the ICUs, and up to 50% of these cases require reintubation within 72 hours [1–5]. Determining the optimal timing of weaning from mechanical ventilation is a challenge for critical care providers [6, 7]. Early weaning is necessary as delayed extubation is associated with an increased risk of ventilator-associated pneumonia (VAP), prolonged ICU stays, and mortality. Premature extubation also carries its problem: the difficulty of reestablishing artificial airways and compromised gas exchange [8, 9].

Many studies have shown that extubation failure increases the mortality rate, length of hospital stays, health care expenses, and ventilator-associated pneumonia [10, 11]. Therefore, identifying the predictors associated with extubation failure (EF) has attracted the interest of clinicians working in ICUs.

Previous studies have found an association between a variety of predictors and extubation failure: the severity of critical illness, old-age patients, co-existing disease, positive fluid balance, and amount of airway secretions, poor cough reflexes, impaired mental status, and unstable hemodynamic parameters [1, 2, 12–17]. Nonetheless, conflicting results are reported depending on the studied population, weaning parameters, trained expertise, and medical resource available in the clinical setup [18–20].

Understanding the patients’ characteristics and factors determining the extubation outcomes will help to develop a prediction tool. Generating predictive tools and utilizing them through area-specific and resource-oriented strategies that address and improve the extubation outcomes would be extremely helpful in the context of limited resources [6, 21, 22].

Different strategies were designed and applied to decrease EF-related morbidity and mortality [23–26]. Despite these efforts in western countries, its incident, predictors, and impacts remain unexplored in the sub-Saharan countries, where determining the extubation outcome is more challenging. To our knowledge, little is known regarding the association between predictors and extubation in ICUs of resource-poor settings.

Therefore, we sought to evaluate the predictors of EF among adult patients receiving mechanical ventilation in ICUs. So, early identification of EF predictors helps in the decision-making process of extubation from MV in ICUs.

## Method and materials

### Study design and patients

This prospective observational study was carried out from December 1, 2020, to November 30, 2021, in St. Paul’s Hospital Millennium Medical College (SPHMMC) among adult patients who received mechanical ventilation in the central ICU. This study was performed per the Declaration of Helsinki Ethical Principles for Medical Research involving human subjects’ protocol. The study was approved by the institution’s Ethical Review Board and informed written consent was secured from the legal guardian of each study participant. Confidentiality was assured throughout the research. This study was retrospectively registered at http://www.researchregistry.com/ with a registry number: researchregistry8173.

**Fig 1:**
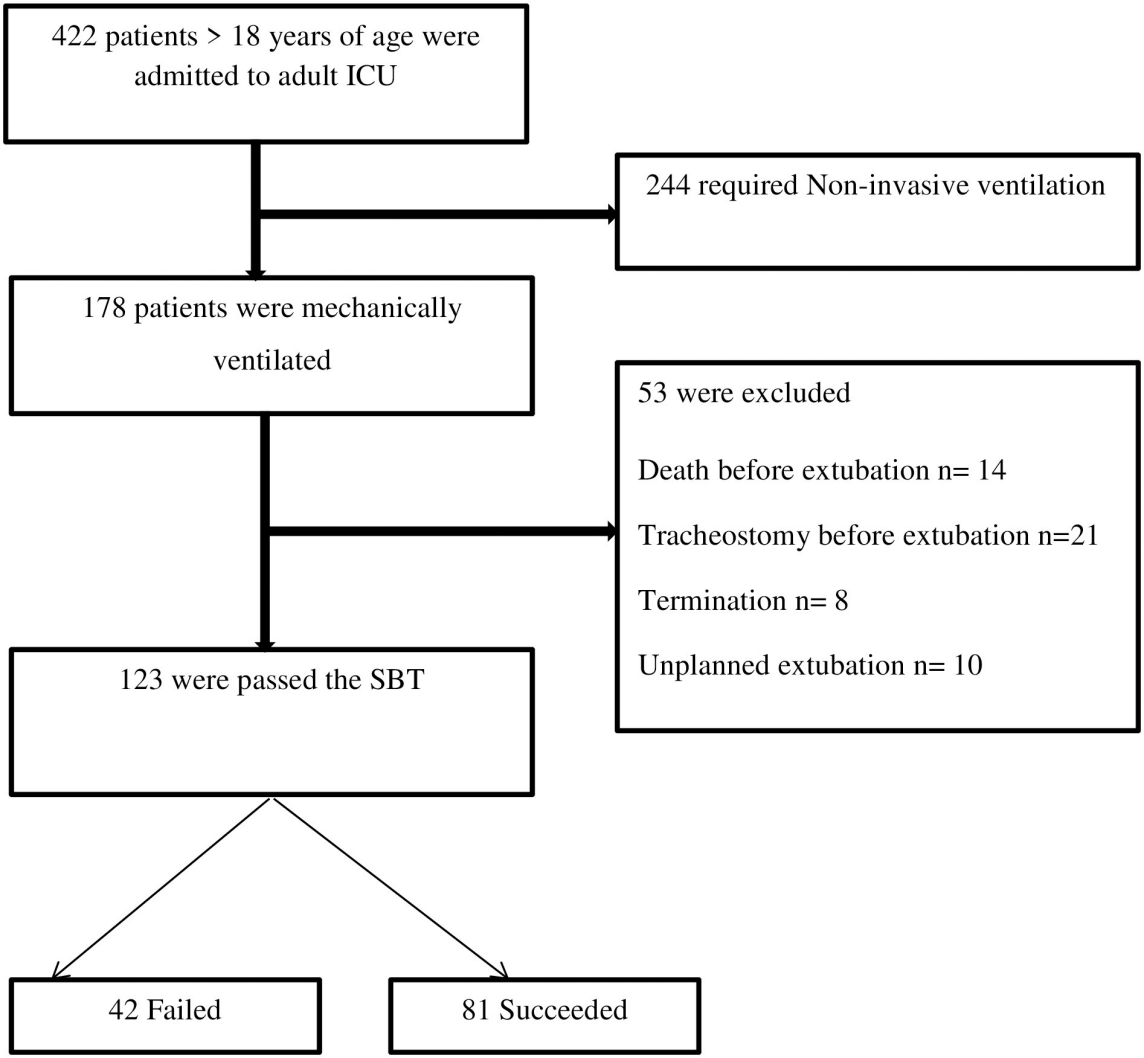
Flow chart of the study participants.

Four hundred twenty-two consecutive patients admitted to the adult ICU during the study period were reviewed. Among them, 123 patients ≥ 18 years of age who received mechanical ventilation for ≥ 48 hours and tolerated spontaneous breathing trials, were included for the final analysis. The exclusion criteria include patients who had undergone a tracheostomy before extubation attempts, died or withdraw support before extubation, and unplanned/self-extubation (Fig 1).

### The process of weaning from mechanical ventilation

Ventilator management was done based on the decision of the attending physician. The anesthesiologist/anesthetist thoroughly evaluated the spontaneous breathing ability of the patient before initiating the SBT. Under close observation for 30–60 minutes, the decision to extubate was made, when the patient met the following criteria; (1) improvement or the resolution of the critical condition requiring mechanical ventilation; (2) sustained pulse oximetry oxygen saturation > 90% with a fraction of inspired oxygen (FIO2) < 0.40 and positive end-expiratory pressure (PEEP) of < 5–7 cm H2O; (3); if the patient regains spontaneous breathing ability and (4) stable hemodynamic status without the use of vasoactive agents [5–7]. Patients who tolerated the SBTs using either pressure support ventilation of 5–10 cmH2O or a T-piece lasting up to 2 hours extubated and supported by a high-flow oxygen mask or nasal cannula capable of delivering a maximum flow rate of up to 60 L/minutes. If patients failed to maintain oxygen saturation were supported by non-invasive positive pressure ventilation (NPPV) and reintubation. Extubation failure is defined as the need for ventilator support (NPPV or reintubation) within 72 hours of planned extubation [8].

In our resource-constrained ICU, the staffing, medical supplies, and overall infrastructures are suboptimal. Currently, the hospital has 392 beds with an annual average of 200,000 patients, a catchment population of more than 5 million, and over 13 departments. The ICU team includes intensivist, anesthesiologists, nurses, general practitioners, and emergency and critical care physicians. There is no intensivist or respiratory therapist in the setup. Nurses and general practitioners are available 24 hours; the attending physician supervises the overall activities in the units. However, most of the nurses and all general practitioners working in the unit didn’t receive any training in emergency and critical care; they were assigned based on a random rotation. The unit is equipped with 10-beds to provide critical care for critically ill medical, obstetric and surgical patients. Despite onsite laboratory tests, radiology, and pharmacy services are not practically available, ICU cases prioritized per the institution’s protocol. The standard monitoring includes non-invasive blood pressure, pulse oximetry (oxygen saturation), and electrocardiogram (ECG). There is no invasive monitoring like arterial blood gas analysis and invasive blood pressure. The unit provides critical care for adult patients who require invasive mechanical ventilation, hemodynamic support, and close monitoring. We used a continuous infusion of propofol or diazepam intermittently for sedation. We also used morphine and rarely ketamine for analgesia. Pain, agitation, and sedation scores were inconsistently assessed and inadequately managed. In the setup, there are no predefined protocols not only for providing analgesics and sedatives but also for providing post-extubation care.

### The collection of data

We collected our data using a checklist prepared based on previous works of literature from the medical charts and direct bedside observation by two trained anesthesiology residents data collectors. Baseline characteristics and clinical profiles before and during the last SBTs were collected on each patient who received mechanical ventilation through bedside observations and by retrieval of relevant data from the medical records.

Baseline characteristics include demographic data, indication for mechanical ventilation (MV), comorbidities, duration of MV, and complications encountered while the patients were on MV from medical records. Before the last SBTs, the cough strength, GCS, amount of endotracheal secretions, and laboratory results were recorded from medical charts and by direct observation accordingly. During the last SBTs, ventilator variables, and vital signs were collected from the monitoring devices and mechanical ventilators.

Concerning GCS, assessment of the verbal response was impossible, as the endotracheal tubes were in the trachea. Therefore, we evaluated eye-opening and best motor responses only in this study, resulting in a maximum score of 10. After passing the spontaneous breathing trials (SBTs) and removal of the endotracheal tube, extubation outcomes whether failed or successful within 72 hours, were recorded.

The primary outcome of this study was extubation outcomes (extubation failure and success) within 72 hours following extubation. The secondary objectives were an identification of potential predictors that determine the extubation outcomes.

### Statistical analysis

We coded, entered, and analyzed the data using SPSS version 26.0 (SPSS Software, CA, and the USA). We performed an analysis of the Mann-Whitney U test for non-normally distributed parametric data. We utilized an analysis of the student’s t-test for normally distributed numeric data. Numeric data were expressed as median (interquartile range) for asymmetric and mean ± SD for symmetric data. We used the Chi-square and Fisher’s exact tests to analyze categorical variables and presented the categorical data as frequency and percentage. All independent variables with a p-value < 0.05 in the univariate analysis were included in the multivariable logistic regression. We checked multi-collinearity using the variation of inflation factor (VIF) and tolerance test. Adjusted odd ratio (AOR) with a 95% confidence interval (CI) and two-sided p-value < 0.05 to measure the strength of association.

### Operational definitions

#### Weaning

Weaning is the process of liberating the patient from mechanical ventilation and the removal of the endotracheal tube.

#### Spontaneous breathing trial (SBT)

A spontaneous breathing trial is a test used to determine the patient’s ability to breathe without the support of a mechanical ventilator.

#### Cough strength

We assessed the strength of the cough, and the patient was given a score based on cough strength [12].

Strong- wetness appears on a piece of paper held 1–2 cm from the end of the endotracheal tube.
Weak- If there is no wetness in the paper

#### Amount of tracheal secretion

We assessed the airway secretions, and gave a score for patients based on suctioning required per hour [27, 28].

Minimal secretions- Suctioning is required every 2–4 h
Moderate secretions- Suctioning required every 1–2 h
Copious secretions- Suctioning is required several times per hour

## Results

During the study period, 422 patients > 18 years of age were admitted to the adult ICU. Among them, 123 patients who met the eligibility criteria were included in the final analysis. About one-third (34.15%) of the patients had developed an extubation failure (EF). The Extubation success and failure groups did not significantly vary by sex, BMI, comorbidity, indications for MV, and complications encountered on MV. However, the EF group had significantly increased mean age [EF vs. ES: 46.1 vs 40.7; p-value <.001] and mean duration of stay on mechanical ventilation [EF vs. ES: 11.9 vs 8.76; p-value <.001] as compared with ES group (Table 1). In extubation success group 13 patients had > 1 comorbidities.

**Table 1:** Baseline characteristics between the extubation success and failure groups.

Demographic data	Failure (n = 42)	Success (n = 81)	P value
Age, years(mean ± SD)	46.1 + 14.52	40.7 + 11.64	**< 0.001**
**Category of age, n (%)**			
< 40	13 (18.84)	56 (81.16)	**< 0.001**
40–60	18 (50)	18 (50)	
> 60	11 (61.12)	7 (38.88)	
**Sex, n (%)**			
Male	33(35.48)	60(64.52)	0.58
Female	9(30)	21(70)	
BMI, kg/m^2^(mean ± SD)	23.2 ± 6.1	22.1 ± 5.2	0.36
**Indication for mechanical ventilation, n (%)**			
Respiratory disease	16(38.1)	23(28.4)	0.664
Postoperative	5(11.9)	12(14.8)	
Altered mentation	16(38.1)	29(35.8)	
Neuromuscular disease	0(0)	3(3.7)	
Trauma	3(7.14)	7(8.65)	
CVS disease	2(4.76)	7(8.65)	
**Comorbidities, n (%)**			
No comorbidities	13(33.34)	16(66.66)	.06
Hypertension	5(31.25)	11(68.75)	.78
DM	3(25)	9(75)	.48
Heart disease	3(21.43)	11(78.57)	.28
Pulmonary disease	6(30)	14(70)	.78
CKD	3(30)	7(70)	.77
Liver disease	2(28.57)	5(71.43)	.79
Stroke	2(22.23)	7(77.77)	.43
Smoking	4(26.67)	11(73.33)	.51
Others	1(20)	4(80)	.32
**MV duration in days (mean ± SD)**	11.9 ± 2.7	8.76 ± 3.35	**<.001**
**Prolonged duration of MV >10 days**	28 (62.23)	17 (37.77)	**<.001**
**Complications encountered during mechanical ventilation, n (%)**			
Overall rate of complications	20(47.62)	41(49.38)	0.85
Ventilator-associated pneumonia	5(31.25)	11(68.75)	0.36
Sepsis	7(43.75)	9(56.25)	0.27
Shock	4(26.67)	11(73.33)	0.36
Acute kidney injury	2(25)	6(75)	0.45
Others	2(40)	3(60)	0.33

SD; standard deviation, DM; diabetes mellitus, CKD; chronic kidney disease, CVS; cardio-vascular system, MV; mechanical ventilation, BMI; body mass index

Concerning clinical profiles before the last spontaneous breathing trial, the EF group had significantly associated with weak cough strength (EF vs ES: 63.16% vs 36.84%), decreased GCS ≤ 8T (EF vs ES: 69.56% vs 30.44%), increased moderately to copious secretions (EF vs ES: 63.16% vs 36.84%), positive fluid balance (EF vs ES: 53.12% vs 46.88%), increased WBC count (EF vs ES: 71.4% vs 27.16%), low albumin (EF vs ES: 18.18% vs 21.44%), and low hemoglobin level (EF vs ES: 10.34 vs 11.39) compared to ES group (Table 2).

**Table 2:** Clinical profiles before the last spontaneous breathing trial between extubation success and extubation failure groups.

Variables	Failure (n = 42)	Success (n = 81)	P value
**Cough strength, n (%)**			
Weak	14 (58.34)	10(41.66)	**<.001**
strong	28 (28.28)	71(71.72)	
**Glasgow coma score, n (%)**			
≤ 8 T	16(69.56)	7(30.44)	**<.001**
9–10 T	26(26)	74(74)	
**Endotracheal secretions, n (%)**			
None to minimal	18 (21.17)	67 (78.83)	**<.001**
Moderate to copious	24 (63.16)	14 (36.84)	
**Fluid balance**	163.9 ± 213.3	9.5 ± 144.1	**<.001**
Positive fluid balance	34(53.12)	30(46.88)	**<.001**
Negative fluid balance	8 (13.56)	51(86.44)	
**Laboratory results**			
WBC count (mean ± SD)	14.66 ± 3.65	12.1 ± 2.94	**<.001**
WBC count > 12,000 /μL	30 (71.4)	22 (27.16)	**<.001**
Albumin, mg/dL (mean ± SD)	18.18 ± 3.64	21.44 ± 4.34	**<.001**
Hemoglobin, g/dL (mean ± SD)	10.34 ± 1.67	11.39 ± 1.57	**.001**
Hemoglobin ≤ 10 g/dL	29 (46.78)	33 (53.32)	**.003**
BUN (mmol/l) (mean ± SD)	10.95 ± 6.72	11.35 ± 8.24	.67
Creatinine (mmol/l) (mean ± SD)	1.06 ± 0. 94	1.09 ± 0.91	.857

WBC; white blood cell, BUN; blood urea nitrogen, SD; standard deviation

Concerning clinical variables during the last SBT, while ventilator-related parameters and vital signs between the two groups were comparable, the frequency of SBTs > 2 times in the EF group was significantly higher (EF vs ES: 54.54 vs 45.46) compared to the ES group (Table 3).

**Table 3:** Characteristics during spontaneous breathing trials between extubation success and extubation failure groups.

Variables	Failure (n = 42)	Success (n = 81)	P value
Frequency of prior SBTs	2.38 ± 0.8	1.63 ± 0.98	**<.0001**
SBT > 2 times, n (%)	18 (54.54)	15 (45.46)	**.004**
Tidal volume, mL	580.6 ± 87	567 ± 84	0.11
Respiratory rate, breath/ minute	20 ± 11	18 ± 8	0.28
RSBI, RR/TV	86.56 ± 36.7	81.2 ± 22.6	0.15
Minute ventilation, L/min	8.2 ± 2.5	7.6 ± 2.2	0.08
PEEP, cm H2O	5.29 ± 0.46	5.21 ± 0.41	0.35
FIO2	41.24 ± 2. 04	40.71 ± 1.6	0.12
SpO2, %	98.59 ± 0.6	98.71 ± 0.5	0.25
Heart rate, beats/min (mean ± SD)	95± 19	93 ± 20	0.14
Systolic BP, mm Hg (mean ± SD)	139 ± 26	136 ± 28	0.23
Diastolic BP, mm Hg (mean ± SD)	86 ± 12	84 ± 10	0.28

SBT; spontaneous breathing trial, FIO2; the fraction of inspired oxygen, RSBI; rapid shallow breathing index, RR; respiratory rate, TV; tidal volume, PEEP; positive end-expiratory pressure, BP; blood pressure, SD; standard deviation

Multiple logistic regression analysis revealed that the predictors significantly associated with EF were prolonged duration of ventilation (>10 Days), age > 60 years, and presence of moderate to copious secretions. However, frequency of SBT, Hemoglobin level, WBC count, Glasgow coma score, fluid balance, and cough strength did not affect the odds of EF despite being significantly different between extubation failure and success groups (Table 4).

**Table 4:** Multivariate logistic regression analysis showing predictors associated with extubation failure.

Variables	Adjusted OR (95% CI)	P-value
SBT > 2 times	3.543 (.913–11.78)	.069
Moderate to copious secretions	3.483 (1.102–11.4)	**.034**
Age > 60 years	4.157 (1.384–12.482)	**.011**
Prolonged duration of ventilation (>10 Days)	4.772 (1.554–14.66)	**.006**
Hemoglobin ≤ 10 g/dL	2.015 (.190–3.32)	.753
WBC count > 12,000/μL	3.296 (.868–12.508)	.080
Albumin < 25 mg/dL	1.081 (.198–5.91)	.928
Weak cough reflex	2.675 (.543–13.171)	.226
Glasgow coma score ≤ 8	1.282 (.269–6.108)	.755
Positive fluid balance	1.964 (.552–6.986)	.297

## Discussion

This study demonstrated that nearly one-third (33.3%) of the extubations failed: even if the extubation is planned and spontaneous breathing trials (SBTs) are successful. Contrary to the present results, numerous studies performed in various intensive care populations reported that the rate of EF ranges between 5% and 20% [1–5]. The significantly increased rate of extubation failure could be explained by the fact that in our low-resource setup there is a lack of predefined post-extubation care protocols such as prophylactic use of corticosteroids in selected patients, chest physiotherapy, and mode of oxygen delivery by identifying patients at high risk. Secondly, despite mixed populations that include medical, surgical, and obstetrics being admitted to our central ICU, multi-disciplinary expertise involvements are rarely practiced. Further, the disproportionately increased ratio of patients relative to nurses’ workload, Inconsistent use of sedatives and analgesics, under-nutrition due to poor feeding, and limited invasive monitoring and laboratory tests might contribute to the increased rate of EF. Developing uniform protocols and guidelines to optimize post-extubation care in selected patients and organizing specialized ICUs with trained staff and well-equipped settings within the compromised resources could help improve extubation outcomes.

Over the last decades, numerous remarkable improvements have been made in the intensive care units (ICUs) globally. However, many ICUs in developing countries are below the standard with a significant limitation of medical resources, ICU bed capacity, trained expertise, and predefined protocols required to provide a standard critical care service [22, 29]. Despite these limitations, establishing reliable tools is becoming even more crucial in attempts to early identify predictors to determine the extubation outcomes, particularly in the most vulnerable patients, without exposing them to unnecessary risks [9].

EF is a potential adverse event in ICUs; once the patients have been liberated from mechanical ventilator. This detrimental adverse event is associated with an increased risk of nosocomial pneumonia, prolonged ICU stays, and increased mortality risk [8–11]. Therefore, a better understanding of the EF predictors is essential to improve extubation outcomes and the overall quality of ICU care [6, 21].

The current study demonstrated that extubation failure was significantly associated with older age > 60 compared to patients aged between 40–60 and < 40. Similar to our findings, studies done by A.-C. Cheng et al [30] and others [1, 31] found older age patients are significantly increased in the EF group compared to the successful extubation after passing SBT. The derangement of an organ’s functional status as age advances could explain the vulnerability of older patients to develop EF. With advanced aging, the organ system undergoes various physiological, anatomical, and immunological changes, making them more vulnerable to multiple organ failures during high-demand states and possible poor outcomes [32].

Our study found that airway secretions were significantly associated with extubation failure. Patients with moderate to copious airway secretions had a > 3-fold risk of developing an extubation failure compared to those with minimal to no airway secretions. This is in line with many studies [11, 13, 16, 27, 28] conducted in different intensive care populations. This could be explained by the fact that an increase in the extent of secretions in the airway contributes to the decrement of mucociliary clearance, promotes accumulation of mucus, and potentially, airway obstruction [33].

Our study also found that the prolonged duration of intubation > 10 days was significantly associated with extubation failure compared with a shorter MV duration. In the same way, many studies reported that longer MV stays significantly increased the risk of developing EF [2, 13, and 34]. Evidence depicted that considering early tracheostomy for those patients anticipated to have prolonged intubation may avert the occurrences of EF [35, 36].

Uses of the Rapid shallow breathing index (RSBI) in ICU are widely practiced: due to its easy technique and avoidance of complex pulmonary mechanics calculation. Evidence shows RSBI is often used to predict extubation outcomes if the primary indication for mechanical ventilation remains a respiratory problem [37, 38]. We evaluated the predictive value of RSBI among 39 patients who indicated for respiratory problems and found that RSBI < 105 L/min predicts 58% of extubation success. However, it is a poor indicator in which nearly two-thirds of cases are non-respiratory indications, as revealed in the current study.

There are some limitations to our study. This observational study was conducted in the resource-constrained clinical settings of a single-center institution with a limited sample size. Therefore, it is difficult to extrapolate the results to the entire clinical setup. The variations in the standard of clinical setup and observational nature of study design resulted in selection bias, in turn; some important predictors like APACHE score, arterial blood gas analysis, cuff leak test, and analgesic/sedative agents that may affect the extubation outcomes were not included in this study. Despite the identified risk factors being relatively similar, further investigation is required whether predictors of extubation failure in low and high resources settings are different. Therefore, further prospective studies in multi-centered hospitals with a large sample size are required to validate our findings.

## Conclusion

The rate of extubation failure was relatively high compared to other studies. Older patients (> 60 years), moderate to copious airway secretions, and duration of intubation (≥10 days) were significant independent predictors of extubation failure. Based on our study findings, we recommend that patients with the identified predictors should be considered in the decision-making process of weaning from the mechanical ventilator.

## Supporting information

S1 Dataset (XLSX)
